# α-Bisabolol, a Dietary Bioactive Terpene Attenuates Oxidative Stress and Inflammation in Colonic Mucosa of Acetic Acid-Induced Colitis in Rats

**DOI:** 10.3390/ijms26178168

**Published:** 2025-08-22

**Authors:** Salim M. A. Bastaki, Naheed Amir, Shreesh Ojha, Ernest Adeghate

**Affiliations:** 1 Department of Pharmacology and Therapeutics, College of Medicine and Health Sciences, UAE University, Al Ain P.O. Box 15551, Abu Dhabi, United Arab Emirates; naheedmiramir@gmail.com (N.A.); shreeshojha@uaeu.ac.ae (S.O.); 2 Department of Anatomy, College of Medicine and Health Sciences, UAE University, Al Ain P.O. Box 15551, Abu Dhabi, United Arab Emirates; eadeghate@uaeu.ac.ae

**Keywords:** acetic acid, α-Bisabolol, inflammation, oxidative stress, phytochemicals, rats

## Abstract

Inflammatory bowel diseases (IBDs), such as ulcerative colitis, and Crohn’s disease are chronic idiopathic inflammatory diseases of the gastrointestinal system involving interaction between genetic and environmental factors mediating the occurrence of oxidative stress and inflammation. There is no permanent cure for IBD except long-term treatment or surgery (resection of the intestine), and the available agents in the long term appear unsatisfactory and elicit numerous adverse effects. To keep the disease in remission, prevent relapses and minimize adverse effects of currently used medicines, novel dietary compounds of natural origin convincingly appear to be one of the important therapeutic strategies for the pharmacological targeting of oxidative stress and inflammation. Therefore, it is imperative to investigate plant-derived dietary agents to overcome the debilitating conditions of IBD. In the present study, the effect of α-Bisabolol (BSB), a dietary bioactive monoterpene commonly found in many edible plants as well as important components of traditional medicines, was investigated in acetic acid (AA)-induced colitis model in rats. BSB was orally administered to Wistar male rats at a dose of 50 mg/kg/day either for 3 days before or 30 min after induction of IBD for 7 days through intrarectal administration of AA. The changes in body weight, macroscopic and microscopic analysis of the colon and calprotectin levels in the colon of rats from different experimental groups were observed on day 0, 2, 4, and 7. The levels of myeloperoxidase (MPO), a marker of neutrophil activation, reduced glutathione (GSH) and malondialdehyde (MDA), a marker of lipid peroxidation, and the levels of pro-inflammatory cytokines were measured. AA caused a significant reduction in body weight and induced macroscopic and microscopic ulcers, along with a significant decline of endogenous antioxidants (superoxide dismutase (SOD), catalase, and GSH), with a concomitant increase in MDA level and MPO activity. BSB significantly improved the AA-induced reduction in body weight, colonic mucosal histology, inhibited MDA formation, and restored antioxidant levels along with a reduction in MPO activity. AA also induced the release of pro-inflammatory cytokines such as interleukin-1 (IL-1), interleukin-23 (IL-23) and tumor necrosis factor-α (TNF-α). Furthermore, AA also increased levels of calprotectin, a protein released by neutrophils under inflammatory conditions of the gastrointestinal tract. BSB treatment significantly reduced the release of calprotectin and pro-inflammatory cytokines. The findings of the present study demonstrate that BSB has the potential to improve disease activity and rescue colonic tissues from damage by inhibiting oxidative stress, lipid peroxidation and inflammation. The findings are suggestive of the benefits of BSB in IBD treatment and substantiate its usefulness in colitis management, along with its gastroprotective effects in gastric ulcer.

## 1. Introduction

Ulcerative colitis (UC) and Crohn’s disease (CD) are the most common idiopathic inflammatory bowel diseases (IBDs), which are characterized by chronic inflammation of the intestine with alternating relapses and remissions and require long-term treatments [1,2]. The etiology of IBD is not fully understood, though it is considered a group of complex heterogeneous gastrointestinal diseases that involves genetic variations, intestinal microbiota, immunological factors, and environmental factors, which interact with an inherent genetic predisposition [3]. The interaction between these factors leads to disruption of intestinal homeostasis, leading to dysregulated inflammatory responses of the gut, and the imbalances between pro- and anti-inflammatory components are among the key causes of intestinal inflammation in IBD [4]. As inflammation is intimately related to the formation of reactive intermediates, including reactive oxygen and nitrogen species, oxidative stress has been proposed as a possible mechanism underlying the pathophysiology of IBD [5]. Oxidative stress is associated with increased production of oxidizing species, along with an increase in inflammation, which damages cell structures by lipid peroxidation, which, in turn, attacks proteins, causing cell apoptosis and necrosis. Thus, inflammation and oxidative stress have been demonstrated to play a key role in pathogenesis, as well as representing an important therapeutic target for UC [5,6,7].

The available therapies for IBD are not very satisfactory for the majority of the patients who require continuous medication to keep the disease under control [8]. In addition, the currently used agents, including amino salicylates, corticosteroids, immunomodulators, and biological agents, often have various side effects with variable responses, which can limit their therapeutic benefits [9]. Until now, there has been no restoration of health apart from surgery (removing a section of the intestine (resection) for intractable colonic inflammation), and no obstructive process is present. Thus, various treatment strategies have shown favorable effects at first for the treatment of colonic inflammation, but prolonged treatments were followed by severe adverse effects (hepatotoxicity, pancreatitis, and increased risk of infection). An ideal agent would prevent relapses and remissions with minimal adverse effects and lesser variation in therapeutic effects [10]. The basis for using many plants in therapeutics is rooted in their traditional use in diet for medicinal benefits since ancient times [11,12]. Thus, in recent years, the use of plant-based drugs as complementary and alternative medicines has gained popularity among patients with IBD, due to their dissatisfaction with existing IBD treatments, as well as demands for other therapies for the management of IBD [13,14]. The use of plant-based therapies is also gaining popularity due to their pharmacological properties, contributing to the attenuation of oxidative stress and inflammation, as well as correcting the nutritional deficits and preventing complications [12,13,14].

In recent years, numerous plants or plant-derived chemicals, natural bioactive compounds known as phytochemicals, also referred to as phytobiotics or phytogenics, have raised interest in patients with IBD, as they are perceived to improve nutritional deficits, prevent and treat complications [15]. These compounds have been shown to ameliorate intestinal inflammation through different molecular pathways, including anti-inflammatory and immunoregulatory mechanisms, antioxidative properties, and modulation of intracellular signaling transduction pathways [11,12,16]. To date, many novel dietary compounds of natural origin, belonging to different classes of phytochemicals, such as alkaloids, saponins, terpenes, glycosides, and flavonoids, have been shown to be effective in experimental models of colitis by targeting oxidative stress and the immune-inflammatory cascade [11,12,16].

Among different classes, the terpenes commonly found in essential oils of edible plants have garnered immense interest due to their favorable pharmacological properties and therapeutic effects in gastrointestinal diseases [17]. One of the bioactive phytochemicals, α-Bisabolol (BSB), a naturally occurring unsaturated sesquiterpene alcohol also known as levomenol, has received attention due to the health benefits associated with its dietary use. It is abundantly found in various edible and ornamental plants, including Candeia (*Ereman thusery-thropappus*), Sage (*Salvia runcinata*), Anyme wood oil (*Myoporum crassifolium*), Germander (*Teucrium* spp.), German chamomile (*Matricaria recutita*), Roman chamomile (*Chamaemelum nobile*), and Negramina (*Siparuna guianensis*). BSB is a common ingredient in beverages, dietary supplements, and herbal medicines [18,19]. Chamomile preparations are used orally for ulcers, insomnia, rheumatic pain, and gastrointestinal disorders in herbal preparations. Bisabolol, whether from a flower, extract, oil, or purified form, is not considered GRAS. However, BSB itself has been deemed safe for use in cosmetics, personal care products, seasoning, or preservative and food flavoring agent [18]. It is also used for the treatment of colic, diarrhea, and other conditions in infants [20] and for the healing of nipple sore in breastfeeding mothers [21]. For a long time, BSB has been used mostly in cosmetic preparations due to its potent skin and wound-healing properties and as a food preservative and additive in humans [22,23].

BSB has shown multiple pharmacological properties, such as anti-inflammatory [20], antioxidant [24], anti-infective [25], antitumor [26,27], anti-nociceptive [28,29], wound-healing [30], and smooth muscle relaxant activity [31]. BSB is useful in inflammatory conditions of the viscera [29], including the heart [19], lungs [32], uterus [33], skin [34], eye [35], and stomach [28,36,37]. Despite its potent role in oxidative stress and inflammation in various organs, there are scant reports available to date on the role of BSB in colonic tissues. BSB is hydrophobic in nature with an XlogP3 value of 3.8 and is one of the bioavailable compounds upon dietary consumption, and it is believed to achieve the required concentration for its therapeutic effects. BSB has been found to improve the antioxidant network (a synergistic combination of antioxidants that protect against free radical damage, keeping oxidative stress at bay) and restore the redox balance by abolishing oxidative stress [38]. The gastroprotective effect of BSB against ethanol, indomethacin, dextran sodium sulphate, aspirin, and diclofenac has been associated with augmentation of antioxidant and anti-inflammatory activity [36,37,39,40,41].

The plant extract containing BSB has been shown to elicit apoptotic effects in colon cancer cells [26]. Given the potent antioxidant and anti-inflammatory properties, as well as the gastroprotective role of BSB and BSB-enriched chamomile [28,36,37,39,42] in gastrointestinal diseases, including gastric ulcer and colon cancer [26,27], there have been few reports on its effect in an animal model of IBD [41]. Therefore, the current research is designed to assess the remedial effects of BSB in the tentative representation of ulcerative colitis. Consequently, the current investigation has focused on the outcome of the effects of BSB in the rat model of IBD induced by acetic acid (AA) and the underlying free radical scavenging and inflammation-reducing mechanisms. In the current investigation, the effects of BSB on apparent comprehensive and invisible minuscule diseases of the colonic mucosa were investigated. The study is novel in demonstrating the role of BSB in AA-induced inflammation of the colon in rats, as evident from its macroscopic and biochemical studies, and there is a strong corroboration from histopathological studies. Additionally, the enzyme activities involved in the formation of free radicals, as well as pro-inflammatory cytokines and calprotectin levels, were investigated in a rat model of IBD induced by AA.

## 2. Results

### 2.1. Effect of α-Bisabolol on Body Weight

Table 1 presents the body weight of rats (n = 6–8) administered BSB orally by gastric gavage at a dose of 50 mg/kg per day for 3 days before and 30 min after IBD induction. The body weight (BW) in control rats (prior to IBD) was 205.17 ± 3.7, 228.80 ± 3.46, 244.50 ± 2.45, and 255.60 ± 1.96 g on days 0, 2, 4, and 7, respectively (Table 1). IBD significantly (*p* < 0.001) decreased body weight from 228.80 ± 3.46 to 206.83 ± 2.99, from 244.50 ± 2.45 to 210.00 ± 4.66, and from 255.60 ± 1.96 to 211.60 ± 2.71 g on days 2, 4, and 7 of IBD. BSB administered to rats 3 days prior to IBD non-significantly increased BW from 206.83 ± 2.99 to 209.13 ± 4.73 g and from 210.00 ± 4.66 to 214.50 ± 4.86 g on days 2 and 4 of IBD, but significantly (*p* < 0.001) from 211.60 ± 2.71 to 241.25 ± 4.76 g on day 7 of IBD (IBD control). BSB, administered after the IBD, at a dose of 50 mg/kg, and continued for 7 days, non-significantly increased the BW from 206.83 ± 2.99 g to 213.50 ± 4.10 g and from 210.00 ± 4.66 g to 213.38 ± 3.98 g on days 2 and 4 of IBD, but significantly (*p* < 0.001, n = 6) from 211.60 ± 2.71 to 243.75 ± 1.75 g (n = 6–8) on day 7 of IBD (Table 1).

### 2.2. Effect of α-Bisabolol on Macroscopic Ulcer Score (MaUS)

**Figure 1:**
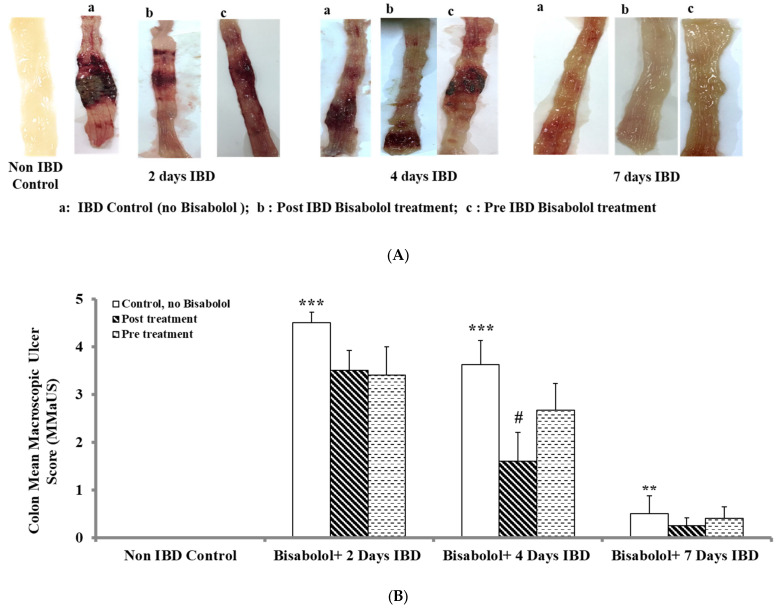
(**A**): Effect of bisabolol on colon macroscopic ulcer score in IBD rat model. (**B**). Effect of bisabolol on colon microscopic ulcer score in a rat model of IBD. Results are mean ± SEM. * Significant difference between bisabolol-treated and non-IBD control. # Significant difference between Bisabolol-treated and IBD control. *** *p* < 0.001; ** *p* < 0.01; # *p* < 0.05.

In the AA-induced IBD rat model, BSB was administered either 3 days before or 30 min after the induction of IBD. The effect of BSB on macroscopic ulcer scores is given in Figure 1A,B. The control rats (untreated, no IBD, 1A) exhibited normal colonic mucosa with no hyperemia or ulceration. IBD rats developed severe hyperemia and ulceration, especially evident at days 2 and 4, but decreased on day 7 of IBD (1A, 1B). A significant (*p* < 0.001) increase in MaUS was observed from 0 (control, no IBD, n = 6) to 4.50 ± 0.22 and 3.63 ± 0.50, after 2, 4, and 7 days of IBD, respectively. BSB was administered 3 days before IBD induction at a dose of 50 mg/kg and non-significantly decreased MaUS on all days of IBD testing. BSB administered 30 min after IBD induction at a dose of 50 mg/kg led to a significant (*p* < 0.05) decrease in MaUS, dropping from 3.63 ± 0.50 to 1.60 ± 0.60 on day 4 of IBD.

### 2.3. Effect of α-Bisabolol on Microscopic Ulcers and Score (MiUS)

**Figure 2:**
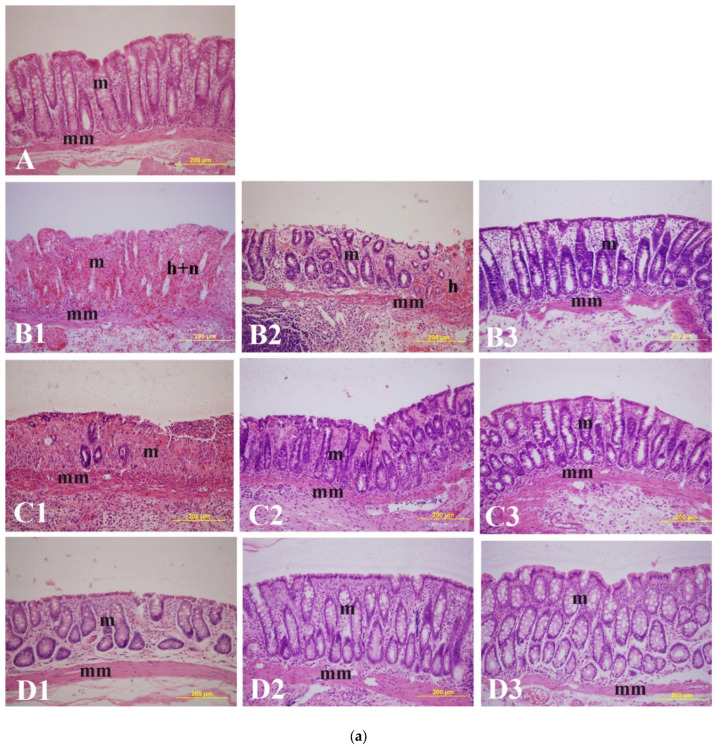

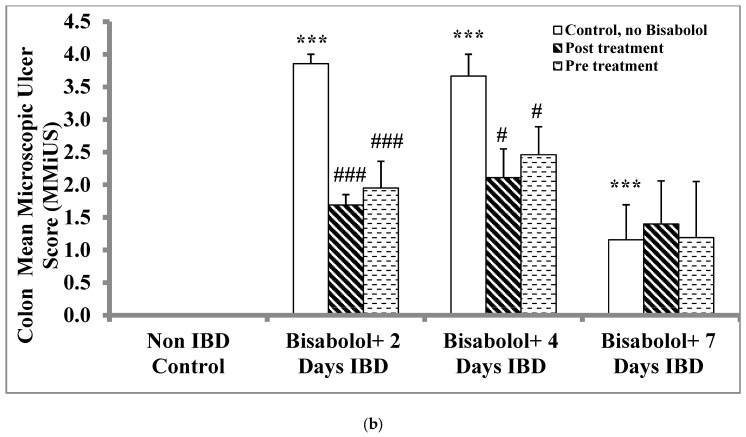
(**a**): Effect of bisabolol on colon microscopic ulcer in IBD rat model. Light micrographs of rat colon show the effects of bisabolol on IBD-induced lesions. (**A**): Naïve (control); (**B1**) 2-day IBD control-no bisabolol, (**B2**): 2-day IBD, post-treated with bisabolol, (**B3**): 2-day IBD (pretreated with bisabolol). (**C1**) 4-day IBD control-no bisabolol, (**C2**): 4-day IBD, post-treated with bisabolol, (**C3**): 4-day IBD (pretreated with bisabolol). (**D1**) 7-day IBD control-no bisabolol, (**D2**): 7-day IBD, post-treated with bisabolol, (**D3**): 7-day IBD (pretreated with bisabolol). (**b**): Effect of bisabolol on microscopic ulcer score in IBD rat model. Data are presented as mean ± SEM. *** Significant difference between bisabolol-treated and IBD-untreated control. ### Significant difference between bisabolol-pre-treated and bisabolol-post-treated rat colon. *** *p* < 0.001 (bisabolol-treated and non IBD control); ### *p* < 0.001 and # *p* < 0.05 (Pre-treated and post-treated versus bisabolol-treated). m = mucosa; mm = muscularis mucosae; h = hemorrhage; h + n = hemorrhage and necrosis. Scale bar = 200 µm.

Histological analysis revealed significant epithelial loss, intense infiltration by inflammatory cells, vasculitis, hemorrhage, and submucosal edema in the colitis group. The influence of BSB on microscopic structure and mean MiUS in the colon mucosa of control, untreated, and treated rats is presented in Figure 2a,b. IBD induction caused a significant (*p* < 0.001) rise in MiUS, increasing from 0 (untreated) to 3.86 ± 0 14 (n = 6), 3.67 ± 0.33 (n = 6), and 1.16 ± 0.54 at days 2, 4, and 7, respectively (Figure 2b. BSB administered at 50 mg/kg 3 days before IBD induction significantly (*p* < 0.05, *p* < 0.001) lowered MiUS on days 2 (3.86 ± 0.14 to 1.95 ± 0.41, n = 6) and 4 (3.67 ± 0.33 to 2.46 ± 0.43, n = 6) but not on day 7 when compared to untreated IBD rats. Furthermore, post-treatment of IBD rats with 50 mg/kg of BSB significantly (*p* < 0.05, *p* < 0.001) reduced MiUS scores on days 2 (3.86 ± 0.14 to 1.69 ± 0.16, n = 6) and 4 (3.67 ± 0.33 to 2.11 ± 0.44, n = 6) in comparison to non-treated controls. This effect was not observed on day 7. The administration of BSB led to a reduction in lymphatic infiltration in the colon of rats treated after IBD induction (Figure 2a). Histopathological examination of rat colon cross-sections showed that the control group exhibited normal-looking mucosal epithelium without signs of necrosis or inflammation (Figure 2a(B3–D3)). In contrast, the AA group displayed diffuse active UC characterized by severe necrosis, hemorrhage, and lymphatic infiltration and inflammation. Pre-treatment with BSB resulted in a partial healing of necrotic areas, showing moderate recovery of epithelial ulcerations along with a reduced degree of necrosis and inflammation. Moreover, histopathological evaluation of the colons from BSB-treated rats demonstrated healing of the 4-amino-3-hydrazino-5-mercapto-1,2,4-trizazole as the chromogen mucosal epithelium, with fewer eroded surfaces and reduced inflammatory edema.

### 2.4. Effect of α-Bisabolol on Myeloperoxidase in Colon

**Figure 3:**
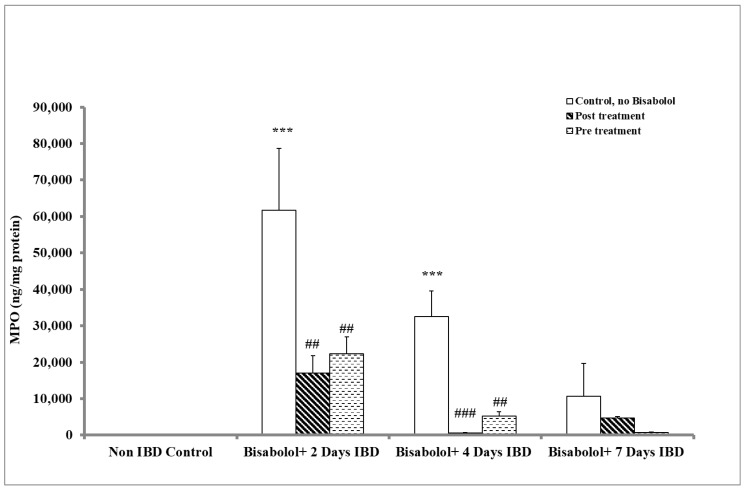
Effect of bisabolol on the activity of myeloperoxidase in colonic tissues of rat model of IBD. The values are expressed as mean ± SEM; *** *p* < 0.001 vs. non-IBD control group: ## *p* < 0.01, ### *p* < 0.001 vs. relative IBD control group.

The activity of myeloperoxidase (MPO) in colonic tissues, which serves as a marker of neutrophil migration to damaged tissue, exhibited a significant increase in the colitis group compared to the control group (*p* < 0.001). To assess the extent of inflammation, the impact of BSB on MPO levels was evaluated in rats that received oral administration for three days either before or 30 min after IBD induction (Figure 3). In the control group (no BSB, no IBD), MPO levels were 113.56 ± 28.85 ng/mg protein. IBD induction led to a significant (*p* < 0.001) increase in MPO levels to 61,739.57 ± 16,904.11 and 32,552.37 ± 7002.41 on days 2 and 4, although levels on day 7 (10,662.75 ± 9043.36 ng/mg protein) were not significantly different from control (IBD, no BSB). Administration of BSB after IBD induction significantly (*p* < 0.01, *p* < 0.001, n = 6) decreased MPO levels from 61,739.57 ± 16,904.11 (no BSB) to 17,037.41 ± 4731.17 ng/mg of protein and from 32,552.37 ± 7002.41 (no BSB) to 577.62 ± 81.21 ng/mg of protein on days 2 and 4 of IBD, respectively. However, BSB had no significant (*p* > 0.05) effect on MPO levels after 7 days of IBD. When BSB was administered 3 days before IBD induction, MPO levels were significantly (*p* < 0.01, n = 6) decreased from 61,739.57 ± 16,904.11 in the untreated IBD group to 22,289.12 ± 4611.25 ng/mL of protein and from 32,552.37 ± 7002.41 to 5241.94 ± 1135.46 ng/mg of protein on days 2 and 4, respectively. BSB caused a non-significant reduction in colon MPO levels on day 7 of IBD.

### 2.5. Effect of α-Bisabolol on Calprotectin Levels in the Colon

**Figure 4:**
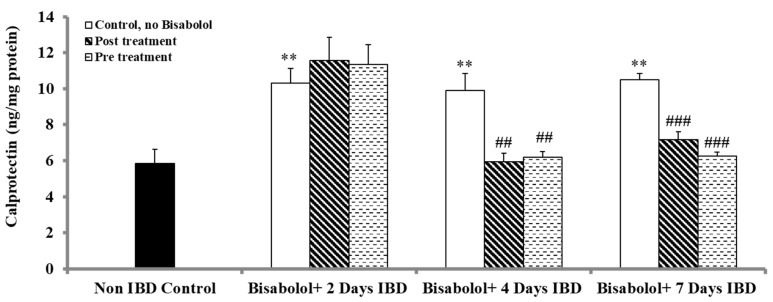
Effect of bisabolol on calprotectin levels. Data are presented as mean ± SEM. * Significant difference between bisabolol-treated and non-IBD control. # Significant difference between bisabolol-treated and IBD control. ** *p* < 0.01, ## *p* < 0.01, ### *p* < 0.001. 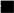 Non-IBD control.

Calprotectin, which is a marker of bowel inflammation in colon tissues, was measured to evaluate the impact of BSB when administered either 3 days before or 30 min after the induction of IBD and treatment was continued for 7 days in rats, as shown in Figure 4. In control animals (no BSB, no IBD), the calprotectin level was 5.85 ± 0.77 ng/mg protein. Induction of IBD caused a significant (*p* < 0.01) increase in calprotectin levels, rising from 5.85 ± 0.77 ng/mg protein (control, no IBD) to 10.31 ± 0.82, 9.90 ± 0.93 and 10.49 ± 0.36 ng/mg of protein after 2, 4 and 7 days of IBD, respectively. Administration of BSB, 30 min after IBD induction, significantly (*p* < 0.01, *p* < 0.001) reduced colon calprotectin levels from 9.90 ± 0.93 to 5.93 ± 0.46 ng/mg of protein and from 10.49 ± 0.36 to 7.16 ± 0.44 ng/mg of protein on days 4 and 7 of IBD, respectively. However, BSB had no effect on colon calprotectin levels (*p* > 0.05) 2 days after IBD induction. BSB administered 3 days before IBD induction, colonic calprotectin levels significantly (*p* < 0.01, *p* < 0.001) decreased from 9.90 ± 0.93 to 6.18 ± 0.34 ng/mg of protein and from 10.49 ± 0.36 ng/mg to 6.24 ± 0.24 ng/mg of protein on days 4 and 7 of IBD. BSB had no effect on colonic calprotectin levels (*p* > 0.05) 2 days after IBD induction.

### 2.6. Effect of α-Bisabolol on Interleukin-1 (IL-1) Levels in Colon

**Figure 5:**
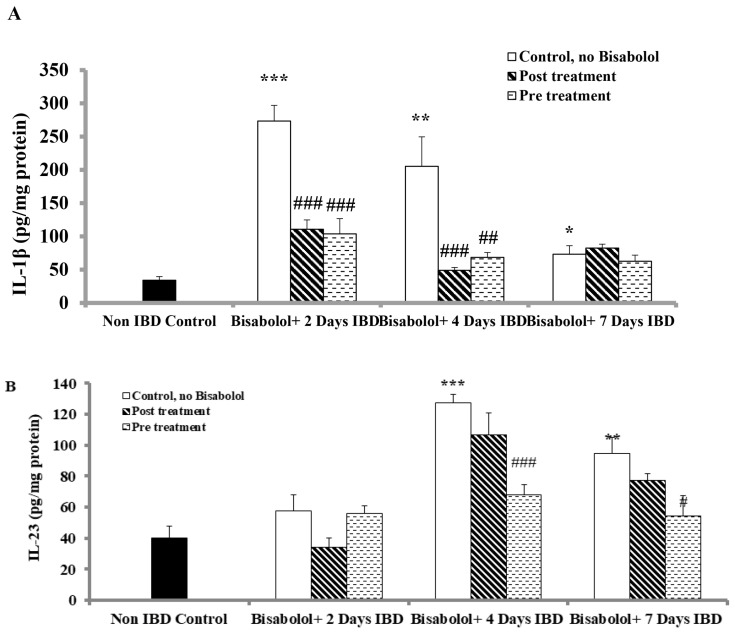
Effect of bisabolol on pro-inflammatory cytokines (**A**) IL-1β, and (**B**) IL-23 in colon. Data are presented as mean ± SEM. * Significant difference between bisabolol-treated and non-IBD control. # Significant difference between bisabolol-treated and IBD control. * *p* < 0.05, ** *p* < 0.01 *** *p* < 0.001; # *p* < 0.05, ## *p* < 0.01, ### *p* < 0.001. 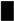 Non-IBD control.

In order to examine the effects of BSB on interleukin-1 (IL-1β), an indicator of inflammation in colon tissues, the rats were treated with BSB either 3 days before or 30 min after the induction of IBD and continued for 7 days, as shown in Figure 5A. In the control group (no IBD, no BSB), the 1L-1β level before IBD was 34.12 ± 5.45 pg/mg of protein. IBD induction significantly (*p* < 0.05, *p*< 0.01, *p* < 0.001, n = 6) raised IL-1β levels to 273.36 ± 23.63, 205.36 ± 44.32, and 73.26 ± 12.78 pg/gm of protein (IBD, no BSB) on days 2, 4, and 7, respectively. However, BSB was administered after IBD induction, and it significantly (*p* < 0.001, n = 6) reduced IL-1β levels from 273.36 ± 23.63 (no BSB, n = 6) to 110.70 ± 14.07 (BSB, n = 6) pg/mg of protein and from 205.36 ± 44.32 (no BSB, n = 6) to 48.99 ± 4.29 (BSB, n = 6) pg/mg of protein on days 2 and 4 days of IBD, respectively. No significant effect of BSB on IL-1β levels (*p* > 0.05) was observed on day 7 of IBD. When BSB was administered 3 days before IBD induction, it significantly (*p* < 0.01, *p* < 0.001, n = 6) lowered the IL-1β levels from 273.36 ± 23.63 (No BSB, n = 6) to 103.81 ± 22.85 pg/mL of protein (IBD, BSB group) and from 205.36 ± 44.32 (No BSB, n = 6) to 68.46 ± 7.32 (BSB, n = 6) pg/mg of protein on days 2 and 4 of IBD, respectively. No significant effect of BSB on IL-1β levels (*p* > 0.05) was observed on day 7 when administered 3 days before IBD induction.

### 2.7. Effect of α-Bisabolol on Interleukin-23 Levels in Colon

To investigate the impact of BSB, levels of interleukin-23 (IL-23), a pro-inflammatory marker in colon tissues, were measured after IBD induction (Figure 5B). In the control groups (no IBD, no BSB), the IL-23 level was 39.95 ± 7.95 pg/mg of protein. IBD induction resulted in a non-significant increase in IL-23 colonic tissue levels on day 2, but a significant (*p* < 0.01, *p* < 0.001) rise was observed on days 4 and 7 of IBD, reaching 127.59 ± 5.46 and 94.54 ± 10.63 pg/mg of protein, respectively (IBD group, without BSB). BSB treatment after IBD induction did not alter IL-23 levels across all tested days. However, when BSB was administered 3 days before IBD, it significantly (*p* < 0.05, *p* > 0.001, n = 6) reduced the IL-23 colonic tissue levels from 127.59 ± 5.46 (no BSB) to 67.71 ± 6.56 pg/mg of protein (BSB, n = 6) on day 4 and from 94.54 ± 10.63 to 54.34 ± 13.27 (n = 6) pg/mg of protein on day 7. BSB had no effect on IL-23 levels on day 2 of IBD induction.

### 2.8. Effect of α-Bisabolol on the Glutathione (GSH) Levels in Colon

**Figure 6:**
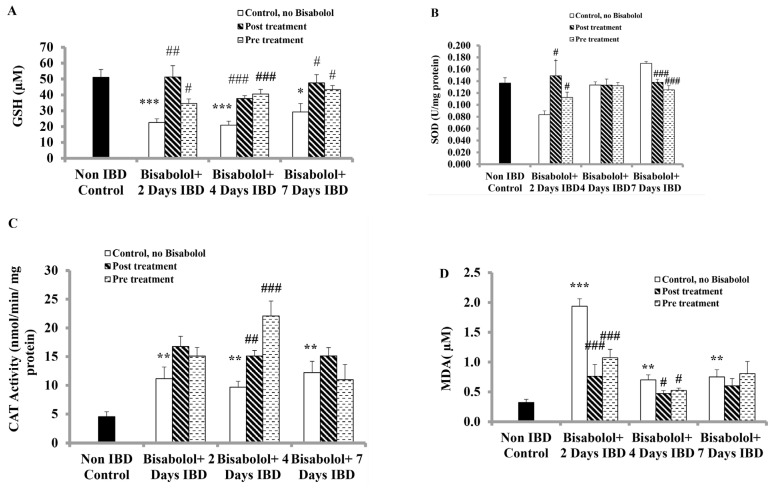
Effect of bisabolol on (**A**) GSH, (**B**) SOD, (**C**) Catalase and (**D**) MDA in colon. Data are presented as mean ± SEM. * Significant difference between bisabolol-treated and non-IBD control. # Significant difference between bisabolol-treated and IBD control. * *p* < 0.05, ** *p* < 0.01 *** *p* < 0.001; # *p* < 0.05, ## *p* < 0.01, ### *p* < 0.001. 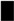 Non-IBD control.

The impact of BSB on GSH levels in colonic tissues of rats following oral administration is illustrated in Figure 6A. In the control group (no IBD, no BSB), colonic GSH levels were 51.06 ± 4.92 μM (n = 6). Induction of IBD reduced GSH levels significantly (*p* < 0.05, *p* > 0.001, n = 6) to 22.52 ± 2.35, 20.94 ± 2.54 and 29.26 ± 5.25 µM on days 2, 4, and 7 of IBD, respectively. When BSB was administered after IBD induction, it significantly (*p* < 0.05, *p* < 0.01, *p* > 0.001, n = 6) increased GSH levels from 22.52 ± 2.35 to 51.25 ± 7.18 µM, from 20.94 ± 2.54 to 37.68 ± 1.82 µM and from 29.26 ± 5.25 to 47.55 ± 5.21 µM on days 2, 4 and 7, respectively. Furthermore, BSB administered orally for 3 days prior to IBD induction significantly (*p* < 0.05, *p* < 0.001, n = 6) increased GSH levels from 22.52 ± 2.35 to 34.56 ± 2.89 µM, from 20.94 ± 2.54 to 40.54 ± 2.87 µM and from 29.26 ± 5.25 to 43.28 ± 2.61 µM on days 2, 4 and 7, respectively.

### 2.9. Effect of α-Bisabolol on Superoxide Dismutase Activity in the Colon

To investigate the effects of Superoxide dismutase (SOD), an antioxidant enzyme that protects against oxidative stress, rats were administered BSB either 3 days before or 30 min after IBD induction (Figure 6B). In the control group (no IBD, no BSB), colonic SOD activity was 0.14 ± 0.01 u/mg of protein. Induction of IBD resulted in a non-significant (*p* > 0.05, n = 6) decrease in mean colonic SOD activity, dropping from 0.14 ± 0.01 to 0.08 ± 0.01 and 013 ± 0.01 u/mg of protein on days 2 and 4, respectively, and recovered to 0.17 ± 0.00 u/mg of protein by day 7. BSB administered after IBD induction significantly (*p* < 0.05, n = 6) increased mean colonic SOD activity from 0.08 ± 0.01 to 0.15 ± 0.03 u/mg of protein on day 2 of IBD, but significantly (*p* > 0.001) decreased from 0.17 ± 0.00 to 0.14 ± 0.01 u/mg of protein on day 7. BSB had no significant effect on SOD activity (*p* > 0.05) on day 4. Pre-treatment with BSB significantly (*p* < 0.05, n = 6) increased colonic SOD activity from 0.08 ± 0.01 to 0.11 ± 0.01 u/mg of protein on day 2, but significantly (*p* > 0.001, n = 6) reduced it from 0.17 ± 0.00 to 0.13 ± 0.01 u/mg of protein on day 7. Pre-treatment with BSB had no significant impact on SOD colonic activity (*p* > 0.01) on day 4.

### 2.10. Effect of α-Bisabolol on Catalase Activity in Colon

Catalase, an antioxidant enzyme that neutralizes hydrogen peroxide and protects against oxidative stress, was measured to assess the effect of BSB on catalase activity when administered either 3 days before or 30 min after inducing IBD in rats (Figure 6C). In the control group (no IBD, no BSB), colonic catalase activity was 4.56 ± 0.84 nmol/min/10 mg tissue. IBD significantly (*p* > 0.01, n = 6) increased catalase activity from 4.56 ± 0.84 (no IBD) to 11.15 ± 2.04, 9.67 ± 1.05 and 12.22 ± 1.95 nmol/min/10 mg tissue of mean colonic catalase activity on days 2, 4 and 7 of IBD, respectively. Post-treatment with BSB had no significant effect on catalase colonic tissue activities on days 2 and 7 but showed a significant (*p* < 0.01, n = 6) increase on day 4. The mean colonic catalase activity rose from 9.67 ± 1.05 to 15.10 ± 0.98 nmol/min/10 mg tissue (IBD + BSB, n = 6). When BSB was given 3 days prior to IBD induction, it significantly (*p* < 0.001, n = 6) increased the catalase colonic tissue activity on day 4 of IBD from 9.67 ± 1.05 (no BSB) to 22.09 ± 2.59 nmol/min/10 mg tissue (n = 6, BSB). BSB had no significant impact on colonic catalase tissue activity (*p* > 0.05) on days 2 and 7.

### 2.11. Effect of α-Bisabolol on Malondialdehyde (MDA) Levels in Colon

Malondialdehyde (MDA), a marker of lipid peroxidation in colon tissues, was assessed to determine the impact of BSB given either 30 min after or 3 days before IBD induction and continued for 7 days in rats, as illustrated in Figure 6D. In the control group (no IBD, no BSB), MDA level was 0.32 ± 0.05 µM/10 mg of colonic tissue. IBD caused a significant (*p* < 0.01, *p* < 0.001, n = 6) increase in MDA levels from 0.32 ± 0.05 to 1.94 ± 0.13, 0.70 ± 0.09, and 0.75 ± 0.12 µM/10 mg of colonic tissue on days 2, 4, and 7, respectively. When BSB was given 30 min post-IBD induction, it significantly (*p* > 0.05, *p* < 0.001, n = 6) lowered MDA levels from 1.94 ± 0.13 to 0.76 ± 0.20 and from 0.70 ± 0.09 to 0.47 ± 0.05 µM/10 mg of colonic tissue on days 2 and 4, respectively. However, it did not influence MDA levels on day 7. Conversely, BSB given prior to IBD significantly (*p* < 0.05, *p* < 0.001, n = 6) reduced MDA levels from 1.94 ± 0.13 to 1.07 ± 0.14 µM and from 0.70 ± 0.09 to 0.52 ± 0.04 µM/10 mg of colonic tissue on days 2 and 4, respectively, but showed no effect on MDA levels on day 7.

## 3. Discussion

The current study’s results reveal that BSB significantly enhanced body weight and colonic length by reducing oxidative stress and inflammation. Moreover, the maintenance of colonic mucosa integrity suggests that BSB has protective and therapeutic roles in AA-induced colitis in rats.

In recent years, there has been an increased interest in studying and identifying the health benefits of dietary plants and plant-derived phytochemicals for their potential in IBD. An increasing number of experimental studies have shown that plants and phytochemicals eliciting antioxidant and anti-inflammatory activity underline the beneficial effects in IBD [12,17,43,44,45,46,47,48]. The natural antioxidant may not only impede the progression of disease but also rescue the patient from the disease. This is the first report to demonstrate the effect of BSB on an experimental colitis model in rats in line with various other terpene compounds, such as geraniol [44], carvacrol [43], menthol [17], nerolidol [43], betulinic acid [44], celastrol [47] and eucalyptol [48], by suppressing inflammatory processes in animal models of IBD. The present findings suggest that BSB could be a potential molecule for nutraceutical use in IBD, as well as for development as a therapeutic candidate.

The rat model of AA-induced colitis used in the present study is a widely used animal model for assessing the efficacy of candidate phytochemicals that target the pathogenesis of inflammation and oxidative stress [49]. This model is associated with enhanced vasopermeability, sustained neutrophil infiltration, and an upregulated inflammatory mediator profile that mimics the pathogenesis of clinical IBD [50]. The findings demonstrated that BSB modulates the mucosal immune system in the context of clinical IBD by affecting epithelial integrity and colon damage through the infiltration of neutrophils and macrophages. The movement of granulocytes and other leukocytes to inflamed mucosa and superficial ulcers leads to excessive production of pro-inflammatory cytokines. The release and induction of pro-inflammatory cytokines arouse an inflammatory state or inflammatory milieu that sustainably affects colon mucosa integrity and influences intestinal epithelial tissues [51]. In the present study, AA administration induced pro-inflammatory cytokines in colon tissues, suggesting the role of inflammation in the pathogenesis of the disease, which is well supported by the histopathology observations depicting epithelial cell necrosis, edema and neutrophil infiltration in the tissue [52]. The body weight of the rats and length of the colon tissue are also considered reliable and sensitive indicators of the severity and extent of inflammatory response [53]. In the present study, improved body weight, colon length and reduced diarrhea following treatment with BSB indicate its protective effect from ulcerative colitis, in support of the potential of other terpene compounds in colitis [17,43,44,45,46,47,48].

In the present study, the administration of AA showed severe colonic inflammation characterized by colon thickening, goblet cell hyperplasia, crypt destruction, ulceration and tissue necrosis associated with inflammatory cell infiltration, as well as depletion of colonic mucus [43]. The protons are liberated by the protonated form of the acid, which is released into space between the cells and causes enormous acidification between the cells, resulting in very large epithelial injury. Therapy with BSB reduced macroscopic and microscopic injuries in the colon of the rats, as noted in histological studies. The likely explanation for the decreased damage is the protection of the epithelial goblet cells, which are in charge of mucin production. Mucins form a barrier, are paramount glycoproteins and protect the bowel against antigens present in the lumen. An important mode of action is played by the vascular system in the operation of protection and repair of gut mucosa [54,55]. It has been demonstrated that a decrease in gastric blood flow increases the severity and area of gastric ulcers after exposure of gastric mucosa to damaging factors [55]. Intestinal mucosal damage in the experimental IBD is convincingly related to both increased free radical production and a low concentration of endogenous antioxidant defense [6,7].

Under normal physiological conditions, the mucosa of the colon contains relatively low levels of endogenous antioxidants [17,56]. Overproduction of ROS perturbs the endogenous defense systems that culminate in the dysregulated ROS production. High levels of ROS lead to oxidative stress and DNA damage due to an imbalance between innate and exogenous antioxidants and ROS [7,57]. It is well known that enzymatic and non-enzymatic antioxidant systems exist to protect tissues from pro-oxidants and, as there is a balance between these systems, the imbalance in colonic milieu has a role in impairing epithelial cell integrity, impeding mucosal recovery and increasing intestinal mucosal permeability [17,53]. This subsequently attenuates the barrier function and host defense to exogenous bacteria and microorganisms following impaired endogenous antioxidant defense system [6,7,57].

SOD, CAT and GPX are endogenous enzymatic antioxidants, whereas GSH is a non-enzymatic antioxidant. These molecules protect cells and organisms from cytotoxic free oxygen radicals [17,43,53]. SOD is among the most important enzymatic antioxidants. Oxygen radicals are converted into O_2_ and H_2_O_2_ by SOD, and the formed H_2_O_2_ is further converted into water by catalases or enzymes of the glutathione redox cycle, a component of the endogenous antioxidant defense system [17,53]. The present study showed that BSB, by improving SOD and catalase levels, significantly prevented excessive free radical generation and reduced the formation of the lipid peroxidation product, MDA, in the colonic tissues of rats administered AA. An endogenous tripeptide antioxidant substrate, GSH plays a critical role in detoxification, facilitates DNA synthesis and repair, promotes recycling of vitamins C and E, inhibits free radical damage, improves the antioxidant activity of vitamin C and enhances the transport of amino acids [58]. It is utilized as a substrate for the activity of the antioxidant enzyme, GPX, which reduces the toxicity of H_2_O_2_ by forming H_2_O and O_2_ molecules. Subsequently, GSH sheds a hydrogen atom, resulting in the formation of oxidized GSH. GSH is formed by Glutathione reductase from oxidized GSH. Declined GSH levels in colon tissues show the development of oxidative stress, and increased GSH levels after therapy with BSB show the antioxidant properties of BSB in colitis. BSB has demonstrated to impede ROS production and increase GSH levels by expanding gastric sulfydryl groups bioavailability, leading to a reduction in gastric oxidative injury induced by ethanol and indomethacin [29,40], as well as in cardiac tissues too [24].

In addition to reduced antioxidant defense, increased formation of lipid peroxides following lipid peroxidation of membranes, characterized by a marked increase in MDA levels in the colon tissue of AA challenged animals, reflects the level of lipid peroxidation in the tissue, hence its common usage as a marker of lipid peroxidation [59]. MDA is one of the end products from the oxidation of polyunsaturated fatty acids, a process that serves as a common marker of oxidative stress alongside MPO. The secretion of MDA occurs as a result of the toxic impact of active free oxygen radicals. These reactive oxygen species (ROS) affect various biomolecules in the organic environment, with membrane lipids, other lipids, proteins and DNA being primary targets. ROS initiate lipid peroxidation by abstracting a hydrogen atom. Previous studies have demonstrated elevated MDA levels in IBD [17,59]. In addition, increased lipid peroxidation has been identified in AA-associated tissue damage, which is in agreement with what was observed in this study. This supports the notion that agents capable of inhibiting the generation of free radicals or enhancing the body’s endogenous enzymatic or non-enzymatic antioxidant defence systems may help alleviate intestinal inflammation and tissue injury in ulcerative colitis [17,45]. In line with this, BSB has been reported to upregulate antioxidant enzymes, increase nitrite content, inhibit lipid peroxidation and decrease the influx of inflammatory cells (neutrophils) in the gastric mucosa. Additional investigations have also confirmed the potent antioxidant and anti-lipoperoxidation activity of BSB [28].

Accumulation of ROS in colon tissues stimulates inflammation responses and secretion of pro-inflammatory cytokines, such as TNF-α, IL-1 and IL-6 [58]. Inflammation of the colonic mucosa is a key feature of colitis, where the role of pro-inflammatory mediators such as IL-1β, IL-6, IL-23 and TNF-α is well established [60,61]. The elevated levels of IL-1β, IL-23 and TNF-α in colon tissues are often used as a biomarker of the severity of colitis [60,61]. IL-1β produced by monocytes and tissue macrophages plays an essential role in the development of local and systemic, as well as acute and chronic, inflammation [60]. It initiates the inflammatory cascade by activating the expression of further pro-inflammatory cytokines, mainly IL-1β, TNF-α and prostaglandins [60,61]. IL-1β is synthesized in an immature form, which is proteolytically processed to its active form by caspase-1 [20]. The release of IL-6 and IL-1 β depends on the critical inflammatory mediator TNF-α, which plays a critical role in stimulating the release of various inflammatory molecules, such as chemokines, cytokines and proteases. This occurs through activation of the canonical NF-κB signaling pathway. NF-κB, a transcription factor sensitive to redox changes, is typically kept inactive by its inhibitor IκB. Its activation is triggered by reactive oxygen species and other signaling cascades, which lead to the phosphorylation and ubiquitination of I_k_B, promoting its degradation [61]. Once activated, NFκB migrates to the nucleus, where its (p65) subunit regulates the transcription of genes associated with immune response and inflammation. Research has shown that BSB (the compound under investigation) possesses anti-inflammatory properties by suppressing the expression of iNOS and COX-2 genes, achieved through the inhibition of NF-κB signaling in RAW264.7 cells [62]. This inhibition results in reduced levels of pro-inflammatory cytokines, indicating a decreased synthesis and release of these mediators, including IL-23, particularly in colonic tissues. The anti-inflammatory activities observed in the present study are in agreement with previous studies wherein BSB showed potent anti-inflammatory activity by inhibiting release and secretion of pro-inflammatory cytokines in heart [19], lung [32], uterus [33], skin [34], gastric [36], colonic [41] and brain [63] tissues. BSB also showed amelioration of inflammatory events in cecal ligation and puncture-induced systemic infection model in mice [25]. BSB showed strong binding affinity to the active site of the pro-inflammatory proteins in a molecular docking study [34].

Neutrophils and macrophages are involved in the infiltration of AA-induced injury of the colon, and the extent of tissue invasion by neutrophil granulocytes is abundantly reflected, as shown by the activity of an enzyme, MPO, mainly found in azurophilic granules of neutrophils with less concentration in monocytes and macrophages [56,58]. MPO is a sensitive indicator of acute intestinal inflammation and serves as a marker of neutrophil infiltration [64]. During inflammation, neutrophils release MPO, which facilitates the production of hypochlorous acid and other free radicals with antimicrobial properties. These free radicals can damage host cells targeting proteins, DNA and lipids [65]. Neutrophils play a significant part in the pathogenesis of IBD, contributing to an increase in reactive oxygen species and superoxide anions. These molecules enhance the production of hydroxyl radicals and peroxides, resulting in tissue damage, mucosal dysfunction and necrosis of colon tissues. Treatment with BSB decreased polymorphonuclear infiltration, evidenced by a significant reduction in MPO activity. BSB has been shown to reduce the MPO activity in the peritoneal fluid of rats with induced peritonitis, as well as in an in vitro assay of neutrophil degranulation [28] and lung [32] and gastric tissues [36], similar to our study findings in colon tissues.

Furthermore, in the present study, administration of BSB resulted in a decrease in calprotectin, demonstrating its beneficial effects on colonic tissues. Calprotectin is a protein occurring in the cytosol of inflammatory cells and is released by the activation of leukocytes. Increased tissue levels of calprotectin have been shown to be associated with the progression of IBD [66]. BSB has shown itself to be considerably safe, with negligible side effects after oral use, which further reveals its usefulness for therapy and nutritional value in humans.

In the present study, rectal administration of AA resulted in reduced colon length and increased tissue ulceration, inflammatory cell infiltration, necrosis and goblet cell hyperplasia, as confirmed by histopathological analysis. These findings, which are characteristic of IBD, align with previous research using a similar animal model [17,43,58]. Treatment with BSB significantly ameliorated the colon’s histoarchitecture by protecting both microscopic and macroscopic structures and averting the depletion of colonic mucus. Additionally, leukocyte infiltration, tissue edema, and injury, which are histological hallmarks of inflammation, were observed to be reduced following pre-treatment with BSB. The present study findings wherein BSB safeguards against AA-induced colitis, are in agreement with earlier findings in its ability to inhibit gastric mucosal damage by reducing mean gastric lesion area [37], reducing the influx of inflammatory cells (neutrophils) in the gastric mucosa [28] and maintaining gastric integrity and attenuating inflammation in gastric tissues [36].

Integrating the anticancer activity in colon cancer cells by suppressing cell growth, cell proliferation and induction of apoptosis without affecting the viability of normal cells [26,67], as well as antiulcer [28,36] and anti-inflammatory potential [36], BSB appears to be a promising molecule for its therapeutic potential in colitis, a risk factor of colon cancer. Additionally, the results of our study also provide scientific validation of traditional claims of plants containing BSB for the management of gastrointestinal diseases [26,67]. The strength is the determination of microscopic and macroscopic parameters and measurement of calprotectin, a marker of colonic inflammation in IBD. There are a few limitations that disease activity index and molecular mechanisms regulating oxidative stress and inflammatory pathways could have been investigated, and many more animal models could be employed. Taken together, the present study results provide a strong basis for further research on BSB as a potential candidate for IBD and molecular mechanisms and offer hope for pharmaceutical development.

## 4. Materials and Methods

### 4.1. Experimental Animals

All animal experiments were approved by the Animal Ethics Committee of the United Arab Emirates University (Animal Ethics Approval No. ERA 10 Oct-2019-6000). The experimental animals were housed and cared for at the Animal Research Facility at the College of Medicine and Health Sciences, United Arab Emirates University, Al Ain, United Arab Emirates. Adult male Wister rats, weighing 225–240 g, were kept in polycarbonate enclosures containing husk bedding, refreshed every 24 h under controlled environment settings appropriate for the vivarium. The standard rodent chow diet was purchased locally, and the animals had access to water ad libitum. Animals were randomly assigned to different groups and housed accordingly for one week prior to the initiation of the experiments. The animals were refrained from eating for 24 h before the initiation of inflammation of the colon, 8 cm adjacent to the anus, under light anesthesia with ether, by giving 1 mL of 4% AA via the rectum for 30 s after overnight fasting. To minimize solution leakage, the rats were placed in the Trendelenburg position during rectal instillation and maintained in this position for 1 min following the procedure. The phosphate-buffered saline (PBS; 1 mL) was used to flush the colon in similar conditions.

### 4.2. Chemicals and Kits

Sodium deoxycholate, BSB, GSH enzyme kinetic assay kit and, if not specified, all other chemicals were bought from Sigma-Aldrich Co. (St. Louis, MO, USA). BSB is identified as CAS Number: 23089-26-1. Molecular Weight: 222.37. EC Number: 208-205-9. MDL number: MFCD03412455. UNSPSC Code: 12352100. PubChem Substance ID: 57647161 was obtained from Sigma-Aldrich Co. (St. Louis, MO, USA). AA was procured from BDH Prolabo (Johannesburg, South Africa). Complete Protease Inhibitor Cocktail and BCA Protein Assay kit were bought from Thermo Fisher Scientific Inc. (Waltham, MA, USA). Rat MPO sandwich ELISA kit was bought from Hycult Biotech (Uden, The Netherlands). Rat IL-23 was purchased from Novus Biologicals, LLC (10771 E Easter Ave, Centennial, CO 80112, USA). The IL-1β Duo-set ELISA kits were procured from R&D Systems (Minneapolis, MN, USA). MDA assay kit was procured from Northwest Life Science Specialties (Vancouver, WA, USA). Calprotectin ELISA kit was procured from MyBioSource Inc. (San Diego, CA, USA)

### 4.3. Experimental Design

The dose of BSB 50 mg/kg was chosen based on a dose-response pilot study in our laboratory and the basis of results of our previously published studies demonstrating its antioxidant and anti-inflammatory properties [19,24]. BSB was administered under two different protocols: (1) a therapeutic regimen in which the outcome of BSB was put to the test for one week after IBD induction, and (2) a preventive regimen, wherein the induction of IBD was performed 3 days after giving BSB and continued for 7 days. In summary, rats undergoing each treatment procedure were split into two groups: those who received BSB preceding (pre-) IBD induction (n = 18, 6 per group) and those administered BSB after IBD induction (post-IBD BSB treated (n = 18, 6 per group)). A dose of 50 mg/kg BSB in 1% carboxymethyl cellulose (CMC) was delivered orally daily, 30 min after IBD was induced (post-treated groups). CMC 1% was administered alone to control animals using the same technique. To examine the protective, as well as therapeutic, part of BSB on IBD, rats were treated with BSB 3 days before IBD induction (pretreated groups). The weight of the animals was taken on days 0, 2, 4 and 7 post-IBD IBD, and the samples of the colon from control, pre- and post-treatment groups were gathered in liquid nitrogen for biochemical analysis and stored in 4% neutral buffered formalin for the histopathological studies.

### 4.4. Assessment of Macroscopic Ulcer Score

To evaluate colonic inflammation both macroscopically and microscopically, histological samples were collected at specific time points (day 0 and after 2, 4, and 7 days of IBD with or without BSB treatment). On the 7th day after IBD induction, the rats were euthanized using cervical dislocation. The colons were then excised approximately 2 cm above the anal margin, opened longitudinally and washed with saline. Macroscopic damage was assessed by a previously described and well-established scoring method, which takes into account the area of inflammation and the presence or absence of ulcers [68]. The point of reference for evaluating macroscopic damage was built on a modified semi quantitative scoring system (taking into account ulcer severity), where features were graded as follows: 0, no ulcer and no inflammation; 1, no ulcer but local hyperemia; 2, ulceration without hyperemia; 3, ulceration and inflammation at a single site only; 4, ulceration and inflammation at two or more sites; 5, ulceration extending more than 2 cm.

### 4.5. Assessment of Microscopic Ulcer Score

After macroscopic observation, samples of the colon were subsequently excised for microscopic observation according to a previously described method [69]. Briefly, colon tissue samples were immersed in 4% formalin in phosphate-buffered saline (PBS) and fixed for one week to preserve tissue morphology. After fixation, the samples were thoroughly washed under running tap water for 2 h to remove any residual formalin before proceeding to dehydration and embedding steps in paraffin wax. Subsequently, xylene was used to deparaffinize the sections of 5 µM of the tissue, stained with hematoxylin-eosin and were viewed microscopically following a previously described and well-established scoring system [70].

### 4.6. Preparation of Colon Tissue Homogenate

In order to open and prepare the colonic tissue for homogenization, an 8 cm longitudinally cut portion of the distal colon was washed with ice-cold PBS. The tissue was then weighed and homogenized using 10 volumes of ice-cold high KCl lysis buffer (10 mM Tris-HCl, pH 8.0, 140 mM NaCl, 300 mM KCl, 1 mM EDTA, 0.5% Triton for X-100 and 0.5% sodium deoxycholate) supplemented with a complete protease inhibitor cocktail. Homogenization was performed using 2.8 mm beads of ceramic with a bead raptor 4 homogenizer (Omni International, Kennesaw, GA, USA). After incubating the homogenates on ice for 30 min, they were centrifuged at 15,000 rpm at 4 °C for an additional 30 min. The supernatant, which resulted from the centrifugation, was stored at −40 °C until ELISA. The protein concentration in each sample was determined by a commercially available BCA kit.

### 4.7. Determination of Myeloperoxidase (MPO)

Myeloperoxidase (MPO) levels were quantified using a sandwich ELISA following the manufacturer’s protocols. Briefly, 100 μL of the standards or samples were added to 96 well microtiter plate, pre-coated with anti-rat MPO antibodies and incubated at room temperature for 1 h. After washing, 100 μL/well biotinylated antibody was added and incubated for another hour. After another wash, Streptavidin–peroxidase conjugate was applied and incubated for 1 h. After a final wash, the TMB-ELISA substrate was added for 30 min, and the enzyme reaction was stopped with oxalic acid. Absorbance was measured at 450 nm using a microplate reader (Tecan Group Ltd., Männedorf, Switzerland). Levels of MPO were expressed as ng per milligram of protein. The Infinite 200 PRO delivers accurate and highly reproducible absorbance readings across a spectral range of 200 to 1000 nm.

### 4.8. Determination of Calprotectin

Calprotectin levels, a marker for colonic inflammation, were estimated in distal colon protein samples using a sandwich ELISA kit (Commercially available from MyBioSource, San Diego, CA, USA). Briefly, 50 µL sample and standards, along with 100 µL HRP, were added to the pre-coated 96-well microtiter plate and incubated at 37 °C for 1 h. Subsequently, after washing, the TMB-ELISA substrate was added and incubated again at 37 °C. After the addition of stop solution, the absorbance was measured at 450 nm using a microplate reader (Tecan Group Ltd., Mannedorf, Switzerland). The levels of calprotectin were expressed as ng per milligram of protein. The Infinite 200 PRO delivers accurate and highly reproducible absorbance readings across a spectral range of 200 to 1000 nm.

### 4.9. Measurements of Reduced Glutathione (GSH), Superoxide Dismutase (SOD) and Catalase

The content of reduced glutathione (GSH) in colon homogenates was quantified using commercially available kits, following the manufacturer’s protocol. Samples and standards were incubated on a coated 96-well microtiter plate for 30 min at 37 °C. Absorbance was recorded at 450 nm using a microplate reader (Tecan Group Ltd., Männedorf, Switzerland), and the results were expressed as µM/mg GSH per mg of tissue (for sensitivity, accuracy, and range of this method, please refer to the microplate reader absorbance spectrum under MPO). Superoxide dismutase (SOD) activity was measured according to the manufacturer’s instructions. This colorimetric assay utilized the tetrazolium salt to detect superoxide radicals generated by xanthine oxidase and hypoxanthine. One unit of SOD activity was defined as the amount of enzyme required to inhibit 50% of the superoxide radical formation. Absorbance was measured at 450 nm by using the Emax Plus microplate reader (Molecular Devices, CA 94089, USA), and results were expressed as units per mg of protein. The Infinite 200 PRO delivers accurate and highly reproducible absorbance readings across a spectral range of 200 to 1000 nm. Catalase (CAT) activity was determined using Catalase Assay Kit based on the enzyme’s peroxidatic function. The assay involved the reaction of the catalase with methanol in the presence of hydrogen peroxide, with the resulting formaldehyde quantified spectrophotometrically at 540 nm. Measurements were performed using Emax Plus microplate reader (Molecular Devices, CA 94089, USA) (See lines 257, 261), employing 4-amino-3-hydrazino-5-mercapto-1,2,4-trizazole as the chromogen. CAT activity in tissue homogenates is expressed as nmol/min/mg protein.

### 4.10. Measurement of Malondialdehyde (MDA)

The measurement of MDA, a product of lipid peroxidation, in the homogenate of the colon from each group was determined using MDA assay kit. The measurement of MDA is according to the manufacturer’s protocol, and the literature survey shows MDA is measured as μM per 10 m of tissue spectroscopically, depending on dilution. The reaction of MDA with thiobarbituric acid (TBA) to form MDA-TBA2 is the most popular method for estimating MDA in biological samples. However, interference can be a significant problem if not dealt with. This assay provides the most precise, highly sensitive and quantitative determination of TBARS, and an efficient means of dealing with elevated backgrounds commonly associated with TBARs reaction. Describing the methodology in brief, the assay is based on the reaction of malondialdehyde (MDA) with thiobarbituric acid (TBA) to form MDA-TBA adduct, which exhibits strong absorbance at 532 nm. In brief, the deproteinated tissue sample was put in 1M phosphoric acid and butylated hydroxytoluene in ethanol, and subsequently, the mixture was heated for 60 min at 60 °C. The mixture was cooled to room temperature and centrifuged at 10,000 rpm for 3 min, and the pink colored supernatant was determined spectroscopically at 532 nm for MDA assay. The concentration of MDA was shown as μM per 10 mg of tissue.

### 4.11. Determination of Pro-Inflammatory Factors IL-1β and IL-23

The enzyme immunoassay of IL-1β in homogenate of the colon was ascertained by making use of commercial sandwich Duoset ELISA kit. Briefly, the microtiter plate wells were coated, in phosphate buffer saline (PBS) (100 µL/well), with respective primary antibody overnight at room temperature, washed with phosphate-buffered saline containing 0.05% Tween (PBST) and then blocked for one hour, with 1% albumin bovine serum in PBS. The plates, after washing, were incubated for 2 h with tissue homogenates and respective standards. After washing with PBST, a detection antibody was added for a period of 2 h. After a wash, 100 µL of HRP was added and kept for 30 min. At that point, TMB-ELISA from (Sigma Chemical Co., St. Louis, MO, USA) was added, and the color intensity was scrutinized at 450 nm with a Tecan microplate reader (Tecan Group Ltd., Männedorf, Switzerland). The enzyme immunoassay of IL-23 in the colon homogenate was measured by using commercial sandwich ELISA kit. Briefly, the pre-coated and pre-blocked microtiter plate wells were incubated for 1.5 h at 37 °C with tissue homogenates and respective standards. After removal of the liquids, a detection antibody was added for a period of 1 h at 37 °C. After a wash, HRP was added and kept for 30 min at 37 °C. At that point, TMB was added and incubated for 15 min at 37 °C, followed by the addition of stop solution and the color intensity was determined at 450 nm with a Tecan microplate reader (Tecan Group Ltd., Männedorf, Switzerland). (See line 234). Pro-inflammatory factor levels were shown as pg per milligram of protein.

### 4.12. Statistical Analysis

SPSS 23.0 software was used to analyze the data statistically. The data is given as mean plus the standard error of the mean (SEM). To determine the significance of the mean between the groups, independent *t*-tests were used to analyze the data. Values of *p* < 0.05 were regarded as significant.

## 5. Conclusions

This study demonstrates the antioxidant and anti-inflammatory effects of BSB in an acute colon inflammation model induced by AA. BSB significantly reduced colonic inflammation, as supported by macroscopic, biochemical and histopathological evaluations. Furthermore, BSB ameliorated the rise in free radicals, pro-inflammatory cytokines and calprotectin levels.

## Figures and Tables

**Table 1:** Effect of bisabolol on mean body weight in a rat model of IBD.

Groups	Day 0	Day 2	Day 4	Day 7
Non-IBD Control	205.17 ± 3.70	228.80 ± 3.46	244.50 ± 2.45	255.60 ± 1.96
IBD control	207.75 ± 2.34	206.83 ± 2.99 ***	210.00 ± 4.66 ***	211.60 ± 2.71 ***
IBD post-treated	201.86 ± 3.08	213.50 ± 4.10	213.38 ± 3.98	243.75 ± 1.75 **^###^**
IBD pretreated	206.43 ± 3.41	209.13 ± 4.73	214.50 ± 4.86	241.25 ± 4.76 **^###^**

Data represent mean body wt. (gm) ± SEM (n = 8). *** *p* < 0.001 vs. non-IBD control group; Significant difference between bisabolol-treated and IBD control, ^###^ *p* < 0.001.

## Data Availability

The data collected has been presented in the manuscript.
